# Physiological Impact of Afterload Reduction on Cardiac Mechanics and Coronary Hemodynamics Following Isosorbide Dinitrate Administration in Ischemic Heart Disease

**DOI:** 10.1007/s12265-021-10112-0

**Published:** 2021-03-15

**Authors:** Tiffany Patterson, Simone Rivolo, Daniel Burkhoff, Jan Schreuder, Natalia Briceno, Christopher Allen, Rupert Williams, Satpal Arri, Kaleab N. Asrress, Jubin Joseph, Hannah Z. R. McConkey, Howard Ellis, Antonis Pavlidis, Brian Clapp, Divaka Perera, Jack Lee, Michael S. Marber, Simon R. Redwood

**Affiliations:** 1 Cardiovascular Division, King’s College London, St. Thomas’ Hospital, Westminster Bridge Road, SE1 7EH London, UK; 2 Department of Imaging Science, St. Thomas’ Hospital, King’s College London, London, UK; 3 Cardiac Research Foundation, New York, NY USA; 4 Thoraxcenter Erasmus University Medical Center, Rotterdam, Netherlands; 5 Cardiothoracic Department, Guy’s and St. Thomas’ NHS Foundation Trust, London, UK

**Keywords:** Coronary artery disease, Pressure-volume loops, Isosorbide dinitrate, physiology

## Abstract

**Abstract:**

Understanding the cardiac-coronary interaction is fundamental to developing treatment strategies for ischemic heart disease. We sought to examine the impact of afterload reduction following isosorbide dinitrate (ISDN) administration on LV properties and coronary hemodynamics to further our understanding of the cardiac-coronary interaction. Novel methodology enabled real-time simultaneous acquisition and analysis of coronary and LV hemodynamics in vivo using coronary pressure-flow wires (used to derive coronary wave energies) and LV pressure-volume loop assessment. ISDN administration resulted in afterload reduction, reduced myocardial demand, and increased mechanical efficiency (all *P*<0.01). Correlations were demonstrated between the forward compression wave (FCW) and arterial elastance (*r*=0.6) following ISDN. In the presence of minimal microvascular resistance, coronary blood flow velocity exhibited an inverse relationship with LV elastance. In summary this study demonstrated a reduction in myocardial demand with ISDN, an inverse relationship between coronary blood flow velocity and LV contraction-relaxation and a direct correlation between FCW and arterial elastance.

**Graphical abstract:**

The pressure volume-loop and corresponding parameters b The pressure volume loop before (solid line) and after (broken line) Isosorbide dintrate

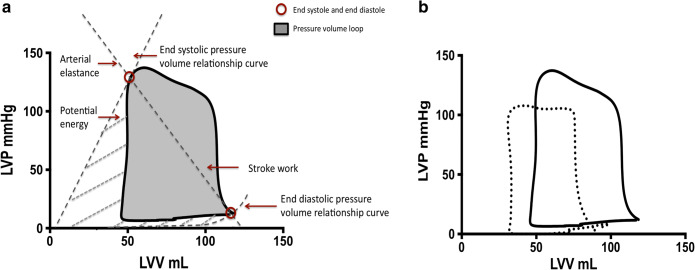

**Supplementary Information:**

The online version contains supplementary material available at 10.1007/s12265-021-10112-0.

## Introduction

**Fig. 1:**
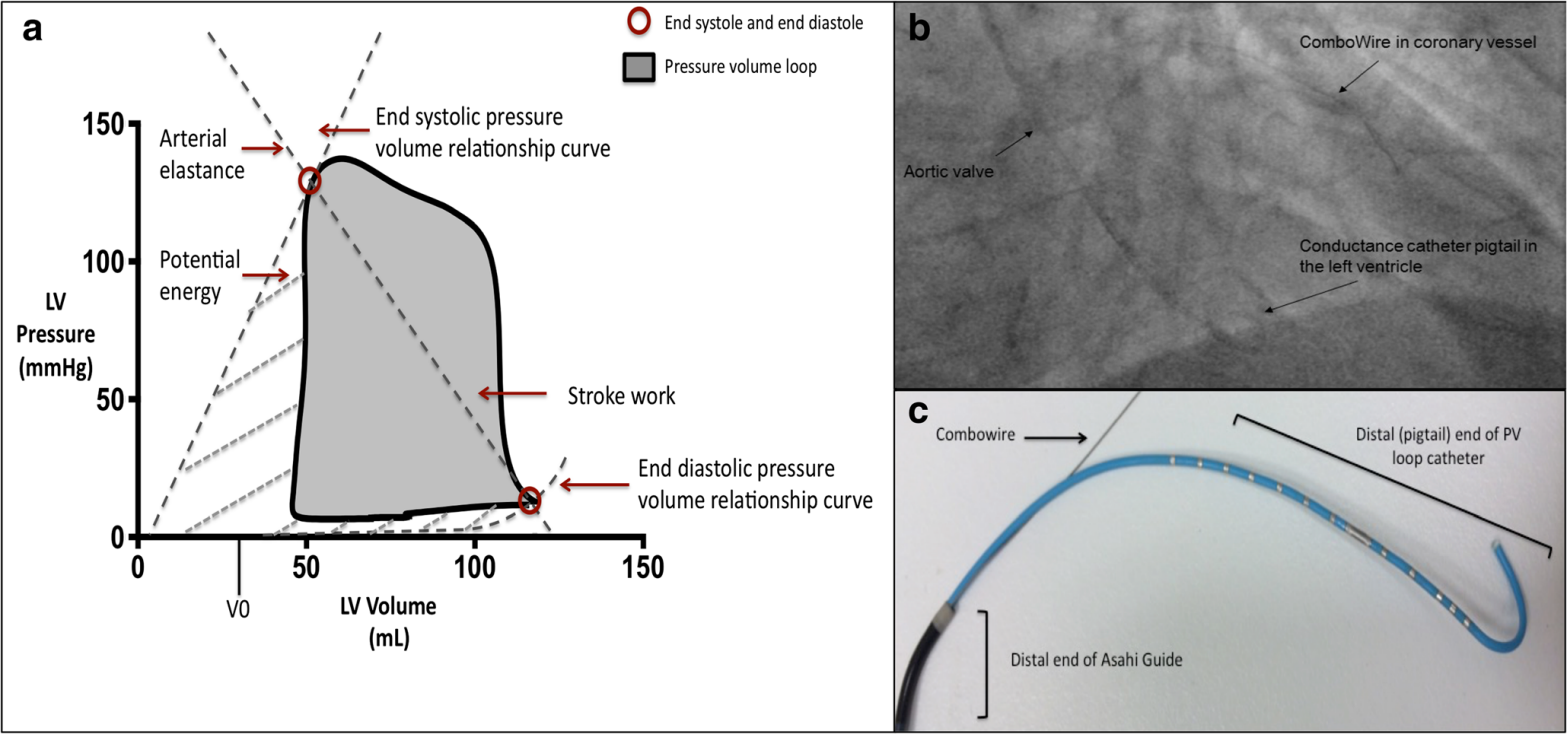
An example of data analysis using the PV loop and acquisition with in-vivo fluoroscopic imaging and ex-vivo demonstration of instrumentation. **a **The pressure-volume (PV) loop and derived measurements. The pressure-volume loop describes a single cardiac cycle by presenting left ventricular pressure as function of volume and facilitates an understanding of cardiac hemodynamics. The end-systolic pressure-volume relationship (ESPVR) line slope (or Ees) represents the load-independent contractile function of the heart. The end-diastolic pressure-volume relationship (EDPVR) line slope (EDPVR) represents the load-independent diastolic properties of the heart. The area of the pressure-volume loop (gray area) represents stroke work (SW), the combination of SW and potential energy (boundary created from the ESPVR and EDPVR lines) represents what is referred to pressure-volume area (PVA or total cardiac work), and arterial elastance is the ratio of end-systolic pressure to stroke volume (Ea). **b** In vivo fluoroscopic image of real-time simultaneous intracoronary and intraventricular data acquisition. Fluoroscopy was used to confirm (PA view) correct positioning of Combowire in the coronary artery (left anterior descending) and conductance catheter (CC) in the apex of the left ventricle. **c** Ex vivo image of Asahi sheathless guide with CC and Combowire exiting the guide catheter

An understanding of the cardiac-coronary interaction is fundamental to the development of treatment strategies in ischemic heart disease [1, 2]. Advances in technology have enabled invasive measurement of left ventricular mechanics to be performed safely in humans. Invasive ventricular pressure-volume (PV) analysis is the current gold standard clinical research method for assessment of left ventricular contractile function and for measuring LV afterload (Fig. 1a) [1, 3]. LV elastance measured using PV analysis has been shown to provide a more accurate measure of contractile properties than conventional indices [4]. Arterial elastance (Ea) provides a more representative measure of LV afterload as it combines both steady and pulsatile forces on the LV and can be derived from the PV loop as the ratio of LV end-systolic pressure to stroke volume [5]. Unlike mean arterial pressure, Ea is not routinely measured in clinical practice, however, this invasive measure of afterload will enable us to monitor the impact of afterload reduction on peripheral and coronary vasculature more accurately. Coronary wave intensity analysis (WIA) derived from coronary pressure and flow velocity can help further identify the transmission of such forces on coronary blood flow by separating the transmission of energy into forward and backward waveforms [6].

The dominant effects of nitrates in the presence of coronary artery disease are believed to be as a result of vasodilatation secondary to afterload reduction [7]. Conclusions are limited however due to the use of surrogate measures of left ventricular mechanics and the assessment of either coronary or left ventricular forces in isolation. To overcome previous limitations, we devised novel methodology, enabling simultaneous invasive assessment of both LV and coronary hemodynamics for the first time. In this physiological study, we sought to examine the impact of afterload reduction following isosorbide dinitrate (ISDN) administration on left ventricular properties and coronary hemodynamics to further our understanding of the cardiac-coronary interaction. The aim of the study was to better understand the relationship between cardiac contraction and coronary blood flow before and after ISDN administration and how the reduction in afterload translates physiologically to anti-anginal properties.

## Methods

### Study Population

This prospective study was conducted at St Thomas Hospital, London, UK. Participants were identified from waiting lists for coronary angiography. Eligibility was confirmed following coronary angiography with identification of coronary artery disease in one or more vessels. This was defined as greater than 50% diameter stenosis in two orthogonal angiographic views. Exclusion criteria included previous coronary artery bypass graft surgery, severe aortic stenosis, and chronic total occlusions that prevented simultaneous LV or coronary hemodynamic measurements. Patients with moderate to severe LV impairment (ejection fraction <45%) were excluded due to a lack of contractile reserve. Participants were requested to stop all oral vasodilator preparations 72 h prior. All patients underwent written, informed consent prior to partaking in the study. This study was deemed safe and received approval from the National Research Ethics Committee (08/H0802/136). In this cohort, 12 patients were necessary to examine the impact of afterload reduction (end-systolic pressure) on cardiac-coronary interaction, providing 25mmHg mean difference (significance defined by two sided alpha 0.05, 80% power). Following interim data review, a further 3 patients were recruited to maximize power due to higher than expected variance about the mean.

### Experimental Protocol and Instrumentation

Left heart catheterization was performed via the right radial artery (6Fr). Following angiographic identification of coronary stenosis, within the same sitting, patients were entered into the study (to avoid multiple procedures), and weight adjusted heparin was administered (70IU/kg) intra-arterially. Thereafter, an 80cm 7.5Fr sheathless guide catheter was used to introduce a dual-sensor pressure-velocity 0.014” coronary guide wire (Combowire, Philips Del Mar) distal to the stenosis in the target coronary artery. The guide catheter was disengaged from the coronary ostium, and a 4Fr conductance catheter (CC) (CD Leycom, The Netherlands) was delivered to the ventricular cavity across the aortic valve via the same guide catheter (Fig. 1b and c). Correct positioning was confirmed by fluoroscopy (Fig. 1b) and conductance signals. Median time from initial diagnostic angiography to completion of instrumentation and initiation of data acquisition was 12 min. At this point, patients were administered either isosorbide dinitrate (ISDN). Baseline measurements including coronary pressure and flow and LV pressure-volume were performed in all patients, and patients acted as their own control. Afterload reduction was performed by manual injection of 1mg (diluted in 10 ml) isosorbide dinitrate through the manifold into the aortic root. Continuous measurements of LV pressure and volume and coronary pressure and flow velocity were performed. Variables were averaged from 5 cardiac cycles. Following the ISDN experimental protocol, physiological assessment of the coronary stenosis was performed with intravenous adenosine (140 mcg/kg/min) peripheral infusion. This was to determine the functional significance of the coronary artery lesion at maximal, steady state hyperemia (maximal coronary blood flow velocity), and patients underwent angioplasty where necessary.

In order to examine the direct relationship between LV contraction-relaxation and coronary flow impediment (compression-relaxation) through a single cardiac cycle, adenosine was also to abolish autoregulatory processes to maximize coronary and capillary vasodilatation. As above continuous recording of resting, simultaneous coronary and LV PV loop measurements were performed at baseline and following the above dosing regimen of IV adenosine administered for 120 s until maximal hyperaemia was achieved. This data was used to generate LV elastance—flow loops (cardiac-coronary interaction plots) and elastance-pressure loops as described below.

### Data Acquisition and Analysis

Simultaneous data acquisition and analysis are described in the supplemental methods [2]. Coronary pressure and flow velocity signals (Combomap system, Volcano Corp) and CC measurements were acquired in real time (Conduct NT, version 3.18.1 CD Leycom). Mean coronary blood flow velocity (U) and distal coronary pressure (Pd) were determined from the Doppler signal and high-fidelity pressure signal, respectively, distal to the coronary stenosis. Figure 1a demonstrates the pressure-volume (PV) loop, and corresponding points on the loop that data were acquired. End-diastole was measured at the peak of the R wave on the ECG, and end-systole was measured at the point of maximum pressure/volume in the cardiac cycle. End-diastolic (EDV) and end-systolic volumes (ESV) were measured from the PV loop. Stroke volume was calculated as the difference in these volumes. End-systolic (ESP) and end-diastolic pressures (EDP) were also measured directly from the PV loop. These baseline measurements were then used to calculate various parameters related to LV performance. The end-systolic pressure-volume relationship curve has previously been shown to be independent of volume loading, within physiological range, and is the most accurate measure of LV contractile function.

This was calculated as the ratio of ESP to ESV, referred to as Ees _(single point, SP)_ [8]. The volume-axis intercept (Vo) of the ESPVR was assumed to be 0 (IVC occlusion was not performed). The slope of the end-diastolic pressure-volume relationship was used to describe LV diastolic function and was calculated using the following equation EDP = An.EDV (Bn), with An = 28.2 mmHg and Bn = 2.79; the values are expressed as the alpha (An) and beta (Bn) coefficients of the equation [9]. LV elastance is the LV pressure-volume relationship over one cardiac cycle. It is a function of LV contractile function over time from relaxation to contraction this forms a bell-shaped curve, where either end (troughs) of the curve represents the end-diastolic pressure-volume relationship (minimum LV pressure) and the peak of the curve represents the end-systolic pressure relationship (ESPVR). In this manuscript, the term LV elastance is used interchangeably to describe contractile function.

LV stroke work which is the external work produced by the ventricle was calculated from the area of the PV loop (Fig. 1a).

Effective arterial elastance (Ea) is the ratio of end-systolic pressure to stroke volume and combines the steady and pulsatile components of left ventricular afterload. Pressure-volume area (PVA) represents total cardiac work and was estimated as the sum of stroke work (SW) and elastic potential energy (Fig. 1a) and has previously been demonstrated to be linearly proportional to myocardial oxygen consumption [10]. LV mechanical efficiency was determined by the ratio of effective stroke work to overall cardiac work (SW/PVA) expressed as a percentage.

For the purposes of analysis, wave intensity analysis (WIA) was performed to establish the temporal relationship between the main coronary wave energies and the cardiac cycle. Wave intensity analysis (WIA) was performed using time derivatives obtained after smoothing the raw signals using an adaptive Savitzky-Golay filter to improve WIA robustness, which has previously been described [11, 12]. Net wave intensity (dI) was performed and normalized to the sampling rate, and separation into forward and backward components was performed using the single-point technique [13]. Baseline pulse wave velocity was implemented for WIA determination during nitrate administration and following adenosine to account for limitations in the single-point technique during hyperemia [14]. We examined the association between afterload and the forward compression wave (FCW), the timing of which has previously been associated with peak aortic pressure, responsible for systolic acceleration of coronary blood flow. We also examined the relationship between diastolic blood pressure and the forward expansion wave which causes late systolic deceleration following a fall in aortic pressure. All four main waves were included in the temporal analysis.

### Statistical Analysis

Quantitative data are expressed as mean (SD) and median (IQR); categorical variables are described as proportions and percentages. Data were assessed for normality of (Gaussian) distribution both graphically and by use of the Shapiro-Wilk test. Statistical comparison of serial hemodynamic measurements (quantitative data) of normal distribution within subjects was performed using paired *t*-tests. Those not of normal distribution were analyzed using Wilcoxon signed rank test. Categorical data were compared by use of the Pearson chi-square test. Correlations were assessed using Pearson’s correlation coefficient. A *P* value of <0.05 was considered statistically significant for all tests. Statistical analysis was performed using SPSS v24.0 (IBM).

## Results

Between December 2013 and May 2016, 15 patients completed the study protocol. Baseline and procedural characteristics of the study population are provided in Table 1. Twenty patients were enrolled, 2 patients had only minor atheroma on coronary angiography, and 3 patients could not complete the study protocol. Baseline measurements were performed in all patients, and patients served as their own control. Eleven of the fifteen patients demonstrated flow-limiting (FL) coronary artery lesions (median FFR 0.72; IQR 0.70 to 0.79). Four of the fifteen patients did not have flow-limiting (NFL) coronary lesions (median FFR 0.88; IQR 0.86 to 0.96) following adenosine administration. All patients responded similarly to ISDN, and there were no differences in baseline characteristics or hemodynamics, details of which and comparison between cohorts (functionally significant versus non-significant lesions) is provided as a table in the supplemental material. Patients were therefore analyzed together and acted as their own control.

**Table 1:** Baseline characteristics of all the study participants (*n*=15 in total) administered ISDN

	Isosorbide dinitrate (15)
Male sex	12 (80)
Age, years	66±14
Height, cm	170±7
BMI kg/m^2^	28.5±3.2
Previous PCI	9 (60)
Previous MI	5 (33.3)
LVEF, %	57 (51 to 63)
Diabetes mellitus	5 (33.3)
Hypertension	10 (66.7)
Hypercholesterolemia	13 (86.7)
Family history	7 (46.7)
Smoking history	11 (73.3)
Current medications	
Βeta-blocker	10 (66.7)
Long-acting nitrate	6 (40)
Statin	12 (80)
ACEi/AIIRB	10 (66.7)
Ca channel antagonist	4 (26.7)
Nicorandil	1 (6.7)
Aspirin	14 (93.3)
Clopidogrel	14 (93.3)
Procedural details	
Diseased vessels	1 (1 to 2)
LAD/Cx/RCA	13/1/2
Duration mins	76 (64 to 91)

Normally distributed continuous data are displayed as mean ± SD, continuous data that are not normally distributed are presented as median (IQR), and categorical data are presented as *n* (%), where *n* is the number of patients in that study group with a certain characteristic. * Indicates *P* value<0.05. *BMI* body mass index, *PCI* percutaneous coronary intervention, *MI* myocardial infarction, *LVEF* left ventricular ejection fraction, *ACEi* angiotensin converting enzyme inhibitor, *AIIRB* angiotensin II receptor blocker, *Pd/Pa* mean distal coronary pressure/man aortic pressure, *LAD* left anterior descending artery, *Cx* circumflex artery, *RCA* right coronary artery

### Left Ventricular Hemodynamics

**Fig. 2:**
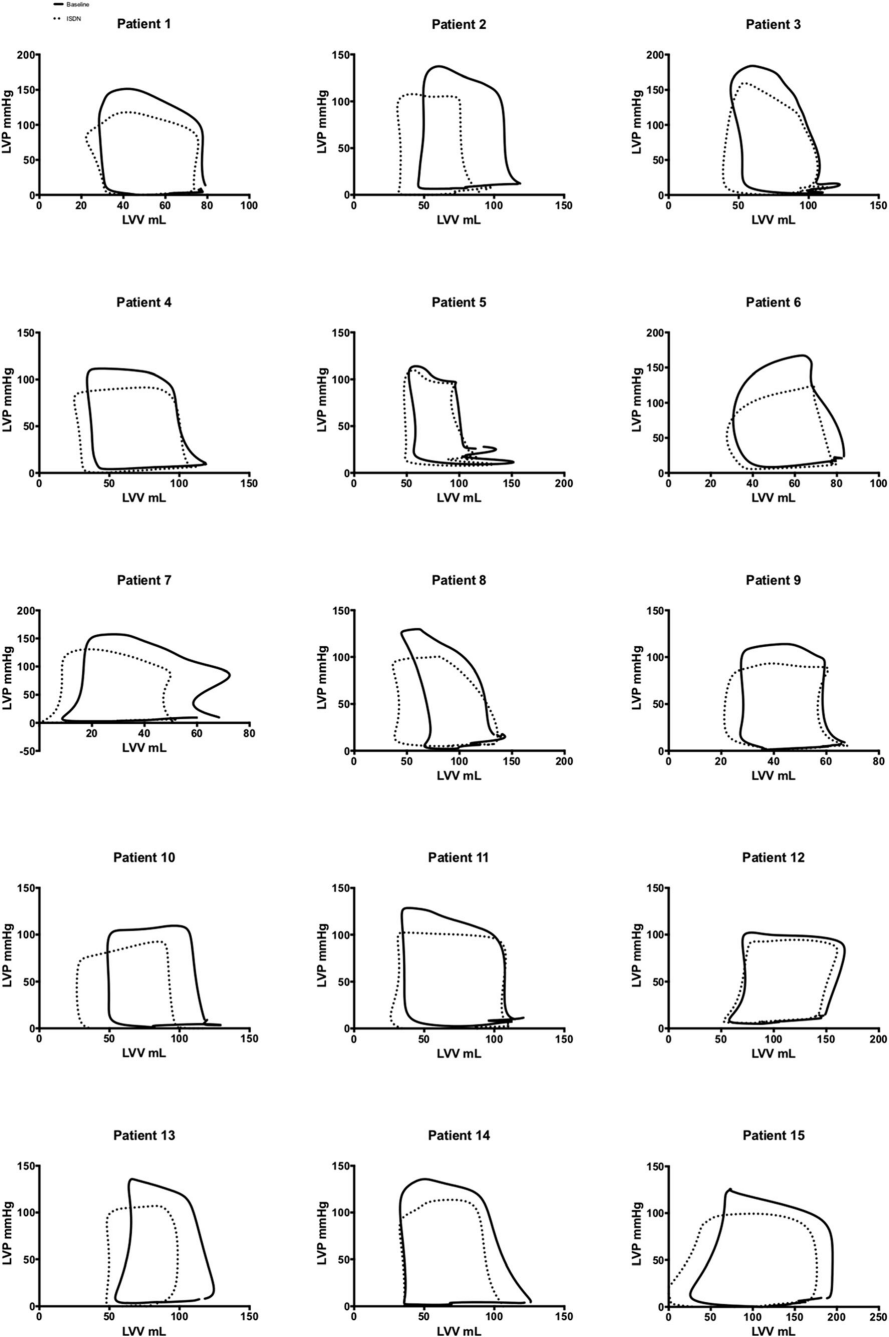
Graphical demonstration of changes induced by ISDN and exercise. **a**–**g** Heart rate, ST segment, power output, EDP, EDP dPdtmax, and coronary flow velocity changes from baseline following administration of ISDN or dynamic exercise. Measurements are at baseline, 50% peak, and peak effect. H LV pressure-volume relations at baseline (black solid line), 50% peak (broken line), and peak exercise (solid red line). Peak exercise effect is associated with increased myocardial demand, stroke volume, and LV pressures. **i** LV pressure-volume relations at baseline (black solid line), 50% peak (broken line), and peak ISDN effect (solid red line). Peak ISDN effect is consistent with a decrease in stroke work, increase in LV efficiency, and reduction in myocardial oxygen consumption

Left ventricular hemodynamic measurements at baseline and following ISDN are presented in Table 2. Graphical representation of LV and coronary hemodynamic measurements are presented in Fig. 2a–g. Administration of intra-arterial ISDN resulted in a leftward, downward shift of the pressure-volume loop compared to baseline (Fig. 2i). Administration of intra-arterial ISDN resulted in a reduction in left ventricular pressures (end-diastolic pressure 8.3±6.4 versus 13.1±6.2mmHg, *P*<0.001; end-systolic pressure 96.0±19.0 versus 122.7±18.0mmHg, *P*<0.001) and left ventricular volumes (end-diastolic volume 102.7±129.5 versus 112.1±32.7mL, *P*=0.01, end-systolic volume 36.1±15.6 versus 44.3±15.3mL, *P*=0.04) compared to baseline. On administration of intra-arterial ISDN, there was a reduction in afterload (arterial elastance 1.3±0.5 versus 1.6±0.5 mmHg/mL, P=0.003) and a reduction in stroke work (6348±2670 versus 7243±2637mmHg.mL, *P*=0.002) and pressure-volume area (1.15±0.4 versus 1.49±0.56J, *P*<0.001) compared to baseline. Administration of intra-arterial ISDN resulted in increased LV mechanical efficiency as a ratio of stroke work to pressure-volume area (73.8±9.0 versus 65.2±6.3%, *P*=0.001). There was also an improvement in the active diastolic properties of the ventricle (Tau 32.5±4.3 versus 34.1±4.6ms; *P*=0.038) but a decrease in magnitude of load-dependent markers of diastolic function (dP/dTmin −906±355 versus −1284±179mmHg/s; *P*=0.011).

**Table 2:** All measured and derived LV and coronary hemodynamic indices and pressure-volume derived indices in participants at baseline and following ISDN

	Baseline	ISDN
LV hemodynamic measurements		
Heart Rate (bpm)	61±10	65±12*
End-diastolic pressure (mmHg)	13.1±6.5	8.3±6.4*
End-systolic Pressure (mmHg)	122.7±18.0	96.0±19.0*
EDV (mL)	112.1±32.7	102.7±29.5*
ESV (mL)	44.3±15.3	36.1±15.6*
Stroke work (mmHg.mL)	7243±2637	6348±2670*
dP/dT (min mmHg/s)	−1284±179	−906±355*
Tau (ms)	34.1±4.6	32.5±4.3*
dP/dTmax (mmHg/s)	1247±206	1227±195
SBP (mmHg)	133±23	108±18*
DBP (mmHg)	99±10	90 ±10*
Beta EDPVR _(SB)_	6.0±0.3	5.8±0.2*
PVA Joules	1.49±0.56	1.15±0.4*
SW:PVA	65.2±6.3	73.8±9.0*
Ees _(SP)_ (mmHg/mL)	2.2±1.0	2.4±1.2
Ea (mmHg/mL)	1.6±0.6	1.3±0.5*
Ea:Ees_(SP)_	0.8±0.2	0.6±0.2*
Coronary hemodynamics and wave energies		
Coronary flow velocity (cm/s)	17.7	20.7
Distal coronary pressure (mmHg)	87.2	76.2*
Microvascular resistance (mmHg/cm/s)	7.4	5.4*
Stenosis resistance (mmHg/cm/s)	3.9	2.4*
Diastolic time fraction	0.64	0.68*
Forward compression wave (J/m^2^/sec^2^)	5822	4568
Forward expansion wave (J/m^2^/sec^2^)	3649	2852
Backward compression wave (J/m^2^/sec^2^)	−7214	−5843
Backward expansion wave (J/m^2^/sec^2^)	−6476	−5882

Patients served as their own control at baseline. Data are displayed as mean ± SD. * Indicates *P* value<0.05. *HR* heart rate, *EDV* end-diastolic volume, *ESV* end-systolic volume, *EF* ejection fraction, *EDP* end-diastolic pressure, *ESP* end-systolic pressure, *E*_*es*_ end-systolic elastance, *Ea* arterial elastance, *SW* stroke work, *PVA* pressure-volume area, *β EDPVR*_*(SB)*_ single beat estimate of the beta coefficient of the end-diastolic pressure-volume relationship curve, *U* mean coronary blood flow velocity, *Pd* mean distal coronary pressure, *DTF* diastolic time fraction, *MVR* microvascular resistance, *SR* stenosis resistance, *BEW* backward expansion wave, *FEW* forward expansion wave

### Coronary Hemodynamics and Wave Intensity Analysis

Prior to ISDN administration median resting PdPa was 0.83 (IQR 0.67 to 0.95). ISDN administration resulted in a significant decrease in distal coronary pressure (76 versus 87mmHg; *P*=0.003), microvascular resistance (5.4 versus 7.4 mmHg/cm/s; *P*<0.001), and stenosis resistance (2.4 versus 3.9 mmHg/cm/s; *P*<0.001) compared to baseline with an increase in diastolic time fraction (0.68 versus 0.64; *P*=0.002) compared to baseline. Wave intensity analysis demonstrated no significant changes in the magnitude of coronary wave energies following administration of intra-arterial ISDN; however, there was a near significant numerical reduction in FCW energy (5822 versus 4568 J/mmHg/s; *P*=0.06).

### Simultaneous Analysis

**Fig. 3:**
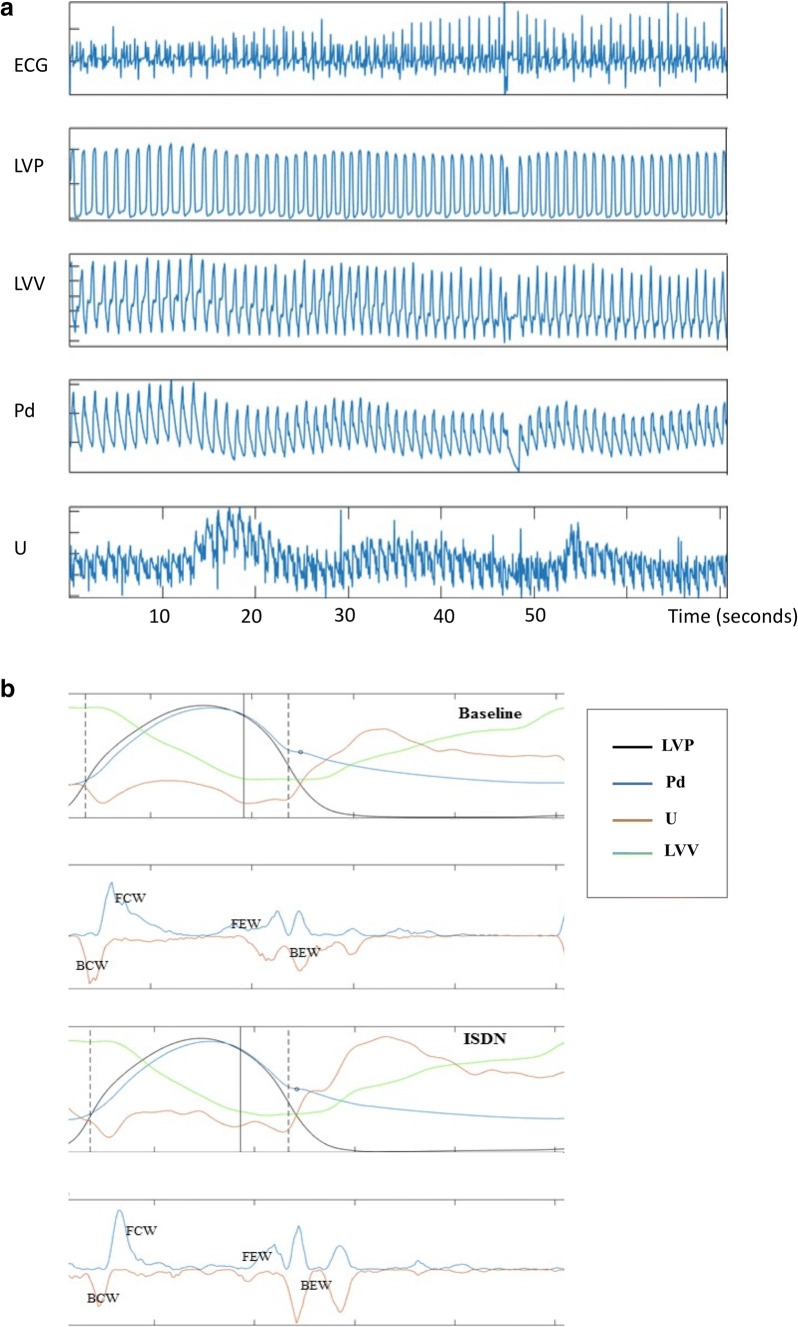
Simultaneous coronary and LV data acquisition. **a** Representative example of simultaneous coronary and LV hemodynamic measurements following intra-arterial ISDN administration as a function of time. From top to bottom, the panels display continuous intra-cardiac ECG recording, left ventricular pressure (LVP), left ventricular volume (LVV), distal coronary pressure (Pd), and mean coronary flow velocity (U). **b** demonstrates simultaneous coronary and LV hemodynamic measurements at baseline and following intra-arterial ISDN administration averaged over one cardiac cycle with coronary wave energies at baseline and following ISDN. **c** demonstrates simultaneous coronary and LV hemodynamic measurements at baseline and during exercise averaged over one cardiac cycle and coronary wave energies at baseline and during dynamic exercise

Figure 3a provides a visual depiction and representative example of simultaneous coronary and LV hemodynamic measurements following intra-arterial ISDN administration as a function of time; a simultaneous drop in LV pressure and subtle increase in coronary blood flow velocity can be seen at 15–20 s. Figure 3b demonstrates the same hemodynamic measurements averaged over one cardiac cycle with coronary wave energies at baseline and following ISDN.

**Fig. 4:**
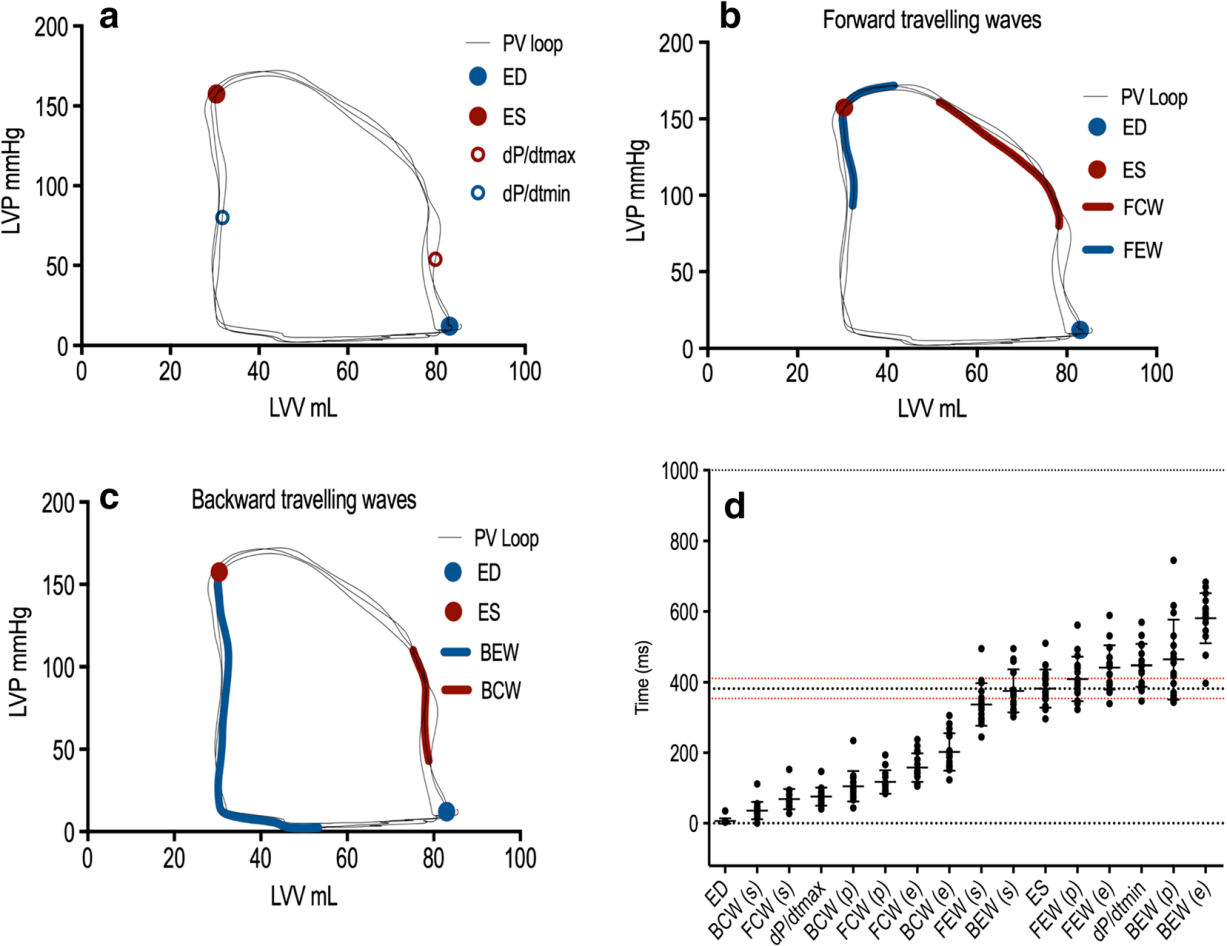
Coronary wave energies superimposed on the pressure-volume (PV) loop. **a** The cardiac cycle depicted as LV pressure (LVP) as function of volume (LVV), the end-diastole (ED), end-systole (ES), and dP/dT max and dP/dTmin (maximal rate of pressure increase and pressure decline, respectively) depicted on the PV loop. **b** The forward travelling compression wave (FCW) in the coronary artery originates when LV pressure equals aortic diastolic pressure (immediately prior to aortic valve opening) and terminates immediately prior to peak LV pressure. The forward expansion wave (FEW) originates when LV pressure equals aortic systolic pressure (peak LV pressure) and terminates during isovolumic relaxation at the point of dP/dT min (maximal LV rate of relaxation). **c** The backward compression wave (BCW) originates during isovolumic contraction and terminates immediately after aortic valve opening (when LV pressure exceeds aortic diastolic pressure). The backward expansion wave (BEW) originates on aortic valve closure, at end-systole, and terminates at minimum LV pressure. **d** Wave generation during the cardiac cycle. The black broken line represents end-systole, with standard deviation (red broken line). *ED* end-diastole, *BCW* backward compression wave, *FCW* forward compression wave, *BEW* backward expansion wave, *ES* end-systole, *FEW* forward expansion wave, *ms* milliseconds, *s* start, *p* peak, *e* end

To examine the association between coronary wave energies and left ventricular pressure-volume, we mapped the timing of the origin and termination of wave energies to the cardiac cycle as measured by the pressure-volume loop during left ventricular contraction and relaxation during resting conditions (Fig. 4). We describe the four main wave energies responsible for acceleration and deceleration of coronary blood flow in relation to the timing of LV contraction and relaxation (Fig. 4b and c). The forward compression wave (FCW) was shown to be generated during isovolumic contraction just prior to aortic valve opening and terminated at peak left ventricular pressure (LV pressure equal to aortic systolic pressure). The backward expansion wave (BEW) was shown to be generated at end-systole (aortic valve closure) and terminated near end-diastole, at the point of minimum left ventricular pressure. The backward compression wave was generated during isovolumic contraction and terminated on aortic valve opening. The forward expansion wave (FEW) was initiated at maximal LV contraction at the point of peak systolic blood pressure and terminated at end-systole (when LV pressure is equal to diastolic blood pressure). The timings of the coronary wave energies including initiation, termination, and peak are presented alongside timings of the major events in the cardiac cycle in Fig. 4d. Wave timings did not change with either exercise or ISDN administration.

**Fig. 5:**
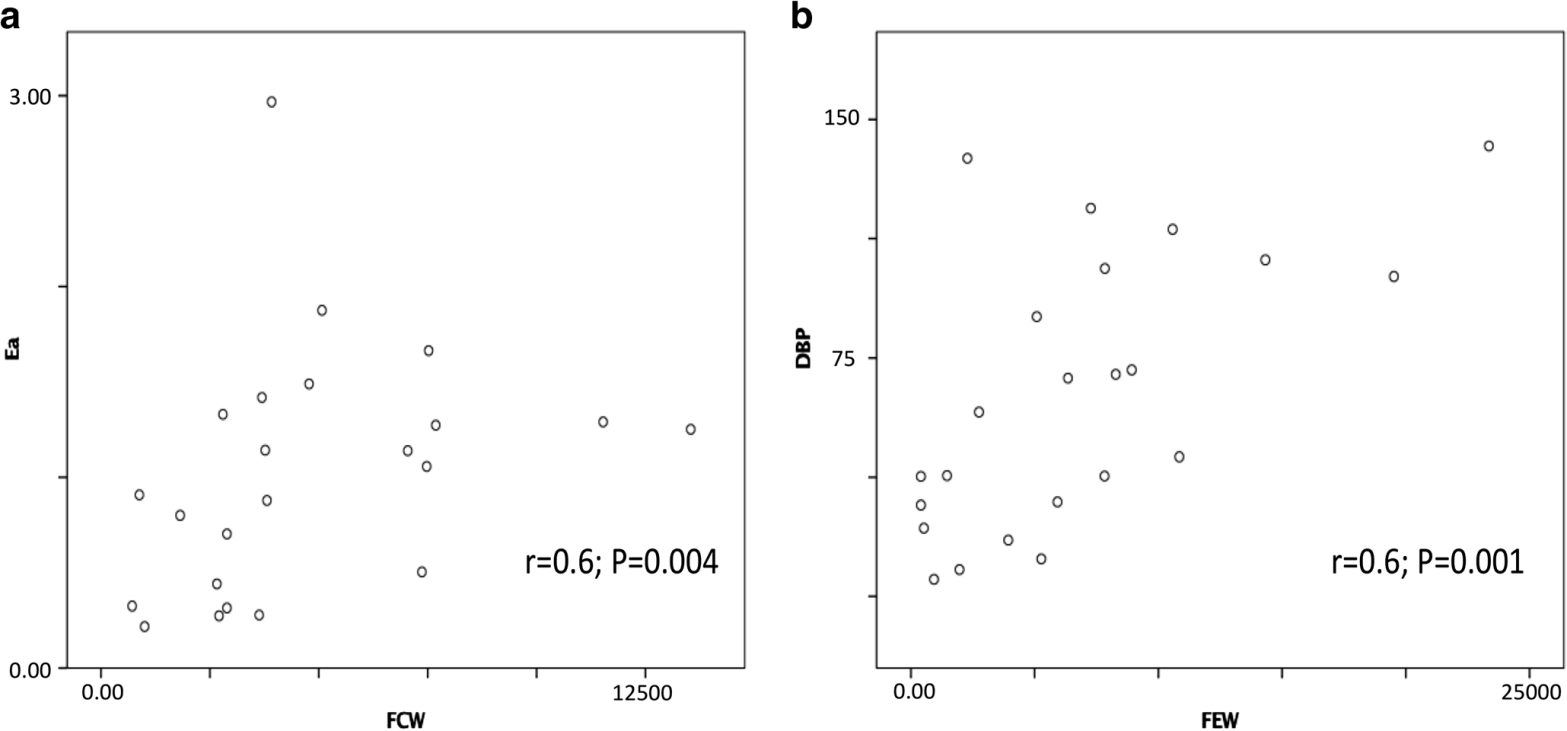
Scatter plot correlations between LV indices and coronary wave energies. **a** Forward compression wave energy and arterial elastance. **b** Forward expansion wave energy and diastolic blood pressure

In light of the above findings and the potential relationship between FCW with aortic pressure, we sought to examine the relationship between the FCW with LV afterload as measured by arterial elastance in the ISDN cohort at baseline. On simultaneous analysis of PV loop and coronary wave energies, afterload as measured by arterial elastance strongly correlated with forward compression wave energy in the coronary artery in all patients at baseline (*r*=0.6, *P*=0.004) and is graphically depicted in Fig. 5a. This relationship was maintained following ISDN administration. Increasing lesions severity was associated with increased magnitude of FCW energy. Furthermore, we examined the relationship between diastolic blood pressure and FEW energy; on simultaneous analysis, we also demonstrated a correlation between FEW magnitude and measured diastolic blood pressure (*r*=0.6, *P*=0.001).

**Fig. 6:**
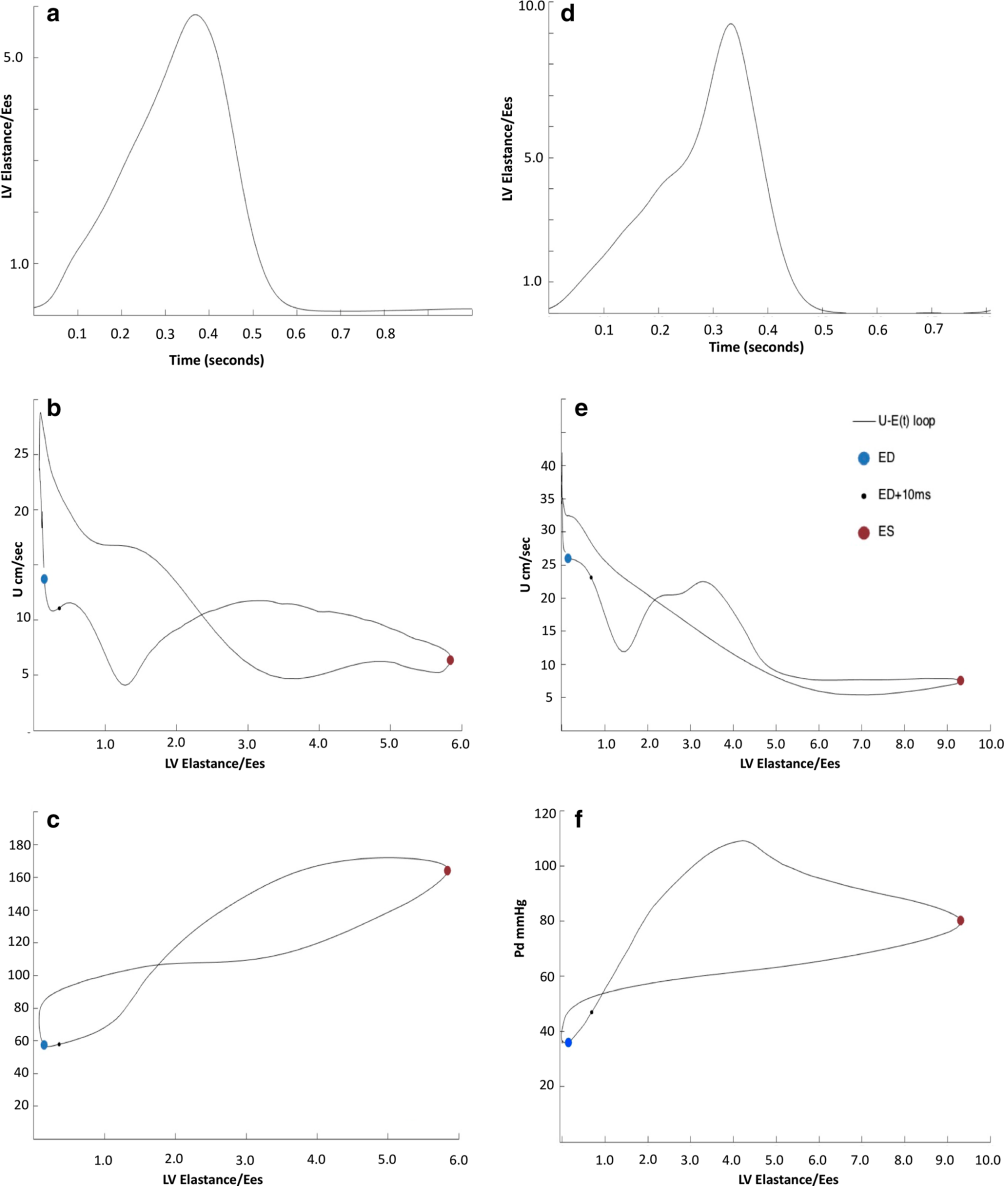
Coronary hemodynamics as a function of LV elastance over a single cardiac cycle. In this diagram, we relate cardiac contraction to coronary blood flow velocity and coronary pressure. **a** to **c** are under resting/baseline conditions. Cardiac contraction over one cardiac cycle is represented as elastance (instantaneous LV pressure/volume). This generates a bell-shaped curve seen in **a**. This starts and ends at end-diastole when LV pressure is minimal and peaks in the center at end-systole. The aim of this plot is to represent LV contraction and relaxation over one cardiac cycle. **b** This is a simultaneous plot of LV elastance (in **a**) against coronary blood flow velocity to demonstrate how LV contraction-relaxation impacts coronary flow velocity; this is performed under resting, baseline conditions. **c** This is a simultaneous plot of LV elastance (from **a**) against distal coronary pressure measured by the Combowire, to demonstrate how LV contraction-relaxation affects coronary artery pressure. **d**–**f** are the same plots after IV adenosine administration; the aim is to abolish autoregulatory processes and maximize vasodilatation to see how this changes the cardiac-coronary interaction D LV elastance plotted against time following adenosine E simultaneous plot of LV elastance (in **a**) against coronary blood flow velocity to demonstrate how LV contraction-relaxation impacts coronary flow velocity following IV adenosine F simultaneous plot of LV elastance (from **a**) against distal coronary pressure measured by the Combowire, to demonstrate how LV contraction-relaxation affects coronary artery pressure. *U* coronary blood flow velocity, *ED* end-diastole, *ED+10* end-diastole + 10ms to identify direction of figure of 8 graph over time, *ES* end-systole

We then examined the association between left ventricular elastance (contractile properties) and coronary blood flow velocity through one cardiac cycle. Figure 6a demonstrates LV elastance over time through one cardiac cycle at baseline. In Fig. 6b, we plotted coronary blood flow velocity against LV elastance at baseline. This demonstrated a figure of eight curve with coronary flow velocity peaking with minimal elastance, and Fig. 6c demonstrates distal coronary pressure as a transmission of LV elastance. Following administration of IV adenosine, to minimize microvascular resistance, there was an increase in LV elastance (Fig. 6d), and in Fig. 6e, we demonstrate a clear inverse exponential relationship between coronary blood flow velocity and LV elastance.

## Discussion

This study methodology enabled simultaneous analysis of coronary hemodynamics and left ventricular pressure-volume parameters. The aim of the study was to better understand the relationship between cardiac contraction and coronary blood flow before and after ISDN administration and how the reduction in afterload translates physiologically to anti-anginal properties. Furthermore, we sought to examine the direct relationship between LV contraction-relaxation and coronary flow (compression-relaxation) during a single cardiac cycle to better understand how the proximity of LV myocardium and coronary arteries impact coronary blood flow. Adenosine was then used to minimize microvascular resistance so that we could identify a direct relation between the two. The main findings of the study are as follows: (1) In the presence of minimal microvascular resistance, coronary blood flow velocity exhibits a predominantly linear inverse relationship with LV contraction through one cardiac cycle. Relative to baseline, the relationship between coronary flow velocity and LV contraction is shifted upward in the presence of minimal MVR; thus for a fixed measure of LV contractility, coronary blood flow velocity is higher. (2) ISDN reduced afterload, preload, and therefore myocardial demand with a subtle increase in coronary flow velocity due to reduction in MVR. (3) Afterload as measured by arterial elastance was shown to strongly correlate with the forward compression wave. (4) The forward compression wave energies derived from invasive coronary hemodynamics can be used as a surrogate measure of arterial elastance without the need for simultaneous invasive LV measurement.

LV elastance is the LV pressure-volume relationship over one cardiac cycle. This is visually depicted in Fig. 6a where either end of the curve represents the end-diastolic pressure-volume relationship (minimum LV pressure) and the peak of the curve represents the end-systolic pressure relationship (ESPVR). Importantly, we were able to demonstrate a relationship between coronary flow velocity and LV elastance (end-systolic pressure-volume relationship over time) for the first time. With autoregulatory processes intact, this relationship is a figure of eight curve. Following administration of IV adenosine to minimize microvascular resistance, we demonstrated a direct linear inverse relationship between coronary flow velocity and LV elastance. This relationship translates clinically to deceleration of coronary flow as the left ventricle contracts until the ventricle reaches the point of maximal contraction. Adenosine also induces maximal vasodilatation (or minimal microvascular resistance), the effect of which is to increase coronary flow, our data demonstrate an upward shift in the cardiac contraction-coronary flow interaction such that for a given measure of LV elastance, coronary flow velocity is increased (Fig. 5e), and despite an increase in contractility (demonstrated with adenosine), coronary blood flow is preserved. This mechanism can be potentially used to explain the cardioprotective effect demonstrated by adenosine and other agents which enhance cardiac contractile function while at the same time driving vasodilatation, such as in the setting of acute heart failure.

We demonstrated that administration of ISDN was associated with a decrease in preload, afterload, total cardiac work, and increased efficiency, thus reducing myocardial demand and therefore the mechanism by which ISDN provides an anti-anginal effect. This study confirmed through simultaneous measurement of coronary and LV hemodynamics that the dominant effects of ISDN were as a result of vasodilatation. ISDN resulted in significant reductions in distal coronary pressure, microvascular resistance, and stenosis resistance with subtle early increase in coronary flow velocity. The latter was likely attributable to wash down the coronary artery from intra-aortic ISDN administration.

Coronary wave intensity analysis generates forward and backward travelling waves [6, 15]. The forward waves are believed to arise upstream, although as a result of left ventricular ejection and relaxation, these waves are believed to be transmitted from the aorta to the coronary artery, thus are forward travelling. Backward travelling waves identified within the coronary artery are thought to originate directly from the myocardium and are generated from the transmission of downstream events in a retrograde manner through the coronary artery. Waves that cause a pressure increase are referred to as compression waves; waves causing a decrease in pressure are referred to as suction or expansion waves. The effect on coronary blood velocity can then be determined by both the origin (forward or backward) and the change in pressure (compression or expansion); for example, an increase in coronary blood velocity can be generated either by a forward travelling compression wave (increase in pressure) or a backward travelling expansion. In this study, administration of ISDN enabled us to vary arterial elastance and demonstrate a direct correlation between the forward compression wave (FCW, flow acceleration arising from aortic pressure) and afterload reduction. Arterial elastance is a superior measure of afterload combining steady and pulsatile forces on the left ventricle [5]; however, this cannot be routine measured in clinical practice without instrumentation of the left ventricle. Therefore, this study demonstrates that using a coronary pressure-flow wire, the derived FCW energy can be used an accurate surrogate measure of afterload and is likely to be superior to measuring non-invasive or invasive arterial pressure alone.

We found that with increasing lesion severity was associated with increased magnitude of FCW energy. Although this initially appears counterintuitive, we propose that because the FCW is responsible for flow acceleration, it would follow that flow velocity would be higher/increased turbulence in the presence of increased stenosis severity. As this a complex interplay between proximal and distal forces, we hypothesis that the presence of progressively severe epicardial stenosis leads to progressive vasodilatation of the coronary microcirculatory bed.

This study examined the timings and origin of wave energies by superimposing them on the cardiac cycle. This provided novel insights into the cardiac-coronary interaction, specifically, that forward travelling coronary waves are dependent on aortic pressure and originate from peripheral vasculature.

## Limitations

This was a single-center study. The population examined were patients with stable single vessel coronary artery disease. A true control group in patients with normal coronary arteries and without risk factors for coronary disease would have provided an interesting comparator. ESPVR was calculated from a single point, not using single beat calculations; V0 was therefore presumed to be 0. Due to the associated technical challenges of simultaneous pressure-volume and coronary pressure and flow measurements, the number of participants was small.

## Conclusions

This study assessed the impact of afterload reduction on LV mechanics with the current gold standard analytical tools. ISDN reduced afterload, preload, and therefore myocardial demand with a subtle increase in coronary flow velocity due to reduction in MVR. Novel findings include improved myocardial efficiency, an enhanced understanding of the cardiac-coronary interaction including the linear inverse relationship between coronary blood flow velocity and LV contraction-relaxation and a direct correlation between FCW and arterial elastance. Afterload as measured by arterial elastance was shown to strongly correlate with the forward compression wave, thus can be used as a surrogate measure of arterial elastance without the need for simultaneous invasive LV measurement. Targeting this wave-elastance relationship could be advantageous in developing treatment strategies in IHD.

## Clinical Relevance

This study demonstrates that the mechanisms of ISDN that contribute to its anti-anginal properties are namely a reduction in cardiac workload and enhance myocardial efficiency. We also demonstrate that forward compression wave energy derived from coronary pressure-flow velocity wire can be used an accurate surrogate measure of afterload and is likely to be superior to measuring arterial pressure in isolation. Adenosine unmasks a linear relationship between LV elastance and coronary blood flow velocity with an upward shift in contractile-flow relations supporting a cardioprotective effect.

## Supplementary Information

ESM 1 (DOCX 17 kb).

## Abbreviations

PV: Pressure-volume
LV: Left ventricle
WIA: Wave intensity analysis
ISDN: Isosorbide dinitrate
CC: Conductance catheter
U: Mean coronary flow velocity
Pd: Distal coronary artery pressure
EDV: End-diastolic volume
ESV: End-systolic volume
EDP: End-diastolic pressure
ESPVR: End-systolic pressure-volume relationship
EDPVR: End-diastolic pressure-volume relationship
Ea: Arterial elastance
PVA: Pressure-volume area
SW: Stroke work
BCW: Backward compression wave
FCW: Forward compression wave
BEW: Backward expansion wave
FEW: Forward expansion wave
dP/dtmax: Maximal rate of pressure increase
dP/dtmin: Maximal rate of pressure decline
