# Supplementary material for: Physiological Impact of Afterload Reduction on Cardiac Mechanics and Coronary Hemodynamics Following Isosorbide Dinitrate Administration in Ischemic Heart Disease
Source: J Cardiovasc Transl Res. 2021 Mar 15;14(5):962–74. doi: 10.1007/s12265-021-10112-0 (PMC8575737; doi:10.1007/s12265-021-10112-0)
Supplement: Supplementary file 1 — (DOCX 17 kb). [file 12265_2021_10112_MOESM1_ESM.docx]

|  | Control | | | Flow limiting | | |
| --- | --- | --- | --- | --- | --- | --- |
|  | Base | ISDN | P value | Baseline | ISDN | P value |
| LV hemodynamic measurements | | | | | | |
| End-diastolic Pressure | 13 ±2.7 | 10.2 ±2.5 | 0.003* | 13±7.2 | 8.5 ±6.9 | 0.003* |
| End-systolic Pressure | 142 ±18 | 112.8± 35 | 0.053 | 118±15.0 | 91.8± 11.5 | <0.001* |
| EDVI | 52±8.4 | 49.5±9.2 | 0.089 | 57.6±17 | 52.7±16 | 0.04* |
| ESVI | 22.6±3.9 | 18.0±8.8 | 0.202 | 22.2±7.7 | 17.9±7.0 | 0.015* |
| Stroke work | 7287 ±1091 | 5972 ±1933 | 0.108 | 7232±2938 | 6442 ±2889 | 0.018* |
| dP/dT min | -1295 ±243 | -1072 ±328 | 0.222 | -1281.5±173 | -865 ±362 | 0.011* |
| Tau | 38 ±5.3 | 35.3 ±3.1 | 0.252 | 33±4.1 | 31.8 ±4.4 | 0.038* |
| dP/dTmax | 1248 ±68.5 | 1243 ±105 | 0.450 | 1247±230 | 1223 ±214 | 0.332 |
| SBP | 157 ±24 | 128 ±28 | 0.030* | 127±19 | 104± 13 | <0.001* |
| DBP | 105 ±6 | 94 ±11 | 0.015* | 96±11 | 90 ±10 | 0.008* |
| Beta EDPVR | 5.9± 1 | 5.9 ±0.6 | 0.021* | 6.0±0.3 | 5.9 ±0.4 | 0.017* |
| PVA | 11493 ±1836 | 8834 ±4121 | 0.106 | 11093±4688 | 8606± 3410 | 0.002* |
| SW:PVA | 63.5 ±2.6 | 70.6 ±12.4 | 0.175 | 65.6±7.0 | 74.6± 8.3 | 0.001* |
| Ees (SP) | 3.4 ±1.0 | 3.3 ±0.4 | 0.871 | 3.1± 1.7 | 3.3± 2.9 | 0.633 |
| Ea | 2.1±0.5 | 1.67± 0.3 | 0.133 | 4.1±0.5 | 1.2 ±0.5 | 0.012* |
| Ea:Ees(SP) | 0.6 ±0.06 | 0.5 ±0.12 | 0.178 | 0.5 ±0.17 | 0.45 ±0.2 | 0.119 |
| Coronary hemodynamics and wave energies | | | | | | |
| Coronary flow velocity | 12.3 | 12.5 | 0.914 | 19.7 | 23.8 | 0.131 |
| Distal coronary pressure | 103 | 87 | 0.143 | 81 | 72 | 0.003* |
| Microvascular resistance | 9.98 | 7.35 | 0.356 | 6.38 | 4.63 | 0.105 |
| Stenosis resistance | 4.6 | 3.1 | 0.324 | 3.63 | 2.01 | 0.005* |
| Diastolic Time Fraction | 0.60 | 0.62 | 0.114 | 0.66 | 0.69 | 0.002* |
| Forward compression wave | 5963 | 5311 | 0.662 | 5769 | 4289 | 0.076 |
| Forward expansion wave | 3617 | 3397 | 0.863 | 3661 | 2648 | 0.262 |
| Backward compression wave | -7861 | -4933 | 0.278 | -6971 | -6184 | 0.623 |
| Backward expansion wave | -8843 | -6243 | 0.203 | -5587 | -5746 | 0.745 |

Supplemental Material

**Table. Cardiac mechanical indices and coronary wave energies in patients at baseline and following ISDN divided into two cohorts: with and without functionally significant coronary disease.**

This supplemental table provides our preliminary analysis which comprised comparison of patient cohorts with and without flow limiting coronary disease at baseline and following ISDN. There were no significant differences between the two groups and both cardiac mechanical indices and coronary indices were similar at baseline in both groups and also responded similarly following ISDN administration. As there were no differences in responses between groups with and without functionally significant coronary disease it was deemed appropriate to group the two together and perform paired analyses to increase numbers and therefore statistical power and sensitivity. Abbreviations: ISDN isosorbide dinitrate, LV Left ventricle, SBP systolic blood pressure, DBP diastolic blood pressure, EDVI end-diastolic volume indexed to BSA, ESVI end-systolic volume indexed to BSA (body surface area), EDPVR end-diastolic pressure volume relationship, PVA pressure volume area, SW stroke work, Ea arterial elastance, Ees LV elastance, SP single point calculation
